# Replacing methyl bromide with a combination of 1,3-dichloropropene and metam sodium for cucumber production in China

**DOI:** 10.1371/journal.pone.0188137

**Published:** 2017-11-16

**Authors:** Liangang Mao, Hongyun Jiang, Lan Zhang, Yanning Zhang, Muhammad Umair Sial, Haitao Yu, Aocheng Cao

**Affiliations:** 1 Department of Pesticides, Institute of Plant Protection, Chinese Academy of Agricultural Sciences, Ministry of Agriculture, Beijing, People’s Republic of China; 2 State Key Laboratory for Biology of Plant Disease and Insect Pests, Beijing, People’s Republic of China; Agroecological Institute, CHINA

## Abstract

The combination of 1,3-dichloropropene (1,3-D) and metam sodium (MNa) is a potential resource to replace methyl bromide (MB) as a soil fumigant. The efficacy of 1,3-D+MNa as a crucial factor to limit soil-borne pests was evaluated in one laboratory experiment and two cucumber greenhouse studies conducted in commercial operations. Laboratory results revealed that 1,3-D and MNa (10+20 mg a.i. kg^−1^ soil) provided the best complementary control of the root-knot nematode, *Fusarium oxysporum* and two species of weed seeds. Greenhouse trials revealed that the blend of 1,3-D and MNa (10+20 g a.i. m^−2^) greatly inhibited the ability of *Meloidogyne incognita* to form root galls. In addition, the number of colony-forming units of *F*. *oxysporum* declined substantially after growth on media, resulting in higher fruit yields and greater economic benefits. The combined use of 1,3-D and MNa exhibited a higher control efficacy than when 1,3-D or MNa was utilized alone. The ability of this chemical combination to control soil-borne organisms did not differ significantly from the MB treatment and maintained high cucumber yields, enhancing the income of the farmers. Compared to the untreated control group, all the chemical treatments prominently improved the control of the pests. These results show that applying a combination of 1,3-D and MNa provides a promising alternative to MB that enables the sustained growth of cucumber production in China.

## Introduction

In North China, cucumber (*Cucumis sativus* L.) is among the most widely cultivated greenhouse vegetables. Soil-borne nematodes, weeds and fungi, exhibit a very strong potential to depress yields in protected cucumbers grown in greenhouses [1,2]. Methyl bromide (MB) has been widely used as a preplant soil fumigant to control soil-borne pathogens, nematodes and weeds in North China. However, MB was restricted for use, except for temporary critical use exemptions (CUEs), in China since January 1, 2015. The removal of MB from agricultural production served as an impetus to identify suitable alternatives that are both economically feasible and do not harm the environment [3].

The current registered chemical MB alternatives for cucumber production in China include metam sodium (MNa) and calcium cyanamide (CC). Sulfuryl fluoride [4], 1,3-dichloropropene (1,3-D) [1,5–7], dazomet (DZ) [7], chloropicrin (Pic) [8], 1,3-D/DZ [7], and 1,3-D/Pic [8] have also been confirmed as chemical alternatives that can be applied in place of MB to protect cucumbers in various countries, including China. However, these active substances do not offer as broad an activity spectrum as that provided by MB [2,9]. To expand the spectrum of action and achieve more effective control over soil-borne pests, combinations of single promising alternative fumigants have become the subject of increasing research in recent years.

Diverse data on the various possible combinations of 1,3-D and Pic [8–15], MNa and Pic [12], 1,3-D and DZ [7,16], dimethyl disulfide (DMDS) and Pic [13,14,17], and DMDS and DZ [18,19] as chemical alternatives to MB have been published. Blends of 1,3-D and methyl isothiocyanate (MITC) in varying concentrations have previously been considered for use in Canada, Europe, and other countries [20]. However, since MITC is highly toxic, its application as part of this combination is prohibited in many countries. Metam sodium (MNa) with its low toxicity, is an important generator of MITC. To attain the maximum outputs and broad-spectrum pest control, the combination of 1,3-D and MNa could serve as a possible alternative. However, more intensive research and development is required to ascertain the potential pest control abilities of this combination. A mixture of 1,3-D and metam in drip fumigation failed to adequately control surplus weeds [21]. This result raises concerns that metam reacts with 1,3-D in aqueous solution, counteracting its effect. In contrast, the sequential application of metam via drip fumigation every two weeks followed by the shank injection of 1,3-D (+ Pic) fumigated the soil as effectively as that with methyl bromide-chloropicrin [22]. The simultaneous application of 1,3-D and MNa accelerated the degradation of 1,3-D, reducing its availability in the soil even though sequential soil treatment or application at varying depths of soil is recommended when 1,3-D and metam are applied together as an admixture [3,23,24]. Previous research has been conducted on the combination of 1,3-D (+ Pic) and MNa in squash (*Cucurbita pepo* L.) [3], strawberry (*Fragaria* × *ananassa* Duchesne) [22,25], and tomato [26]. However, little information has been reported on the combination of 1,3-D and MNa for use on cucumbers.

The purpose of this study was to investigate and validate the efficacy of a blend of 1,3-D and MNa on nematodes (*Meloidogyne incognita*), soil-borne fungi (*Fusarium oxysporum*) and weed seeds (*Abutilon theophrasti* and *Digitaria sanguinalis*) in the laboratory. Two greenhouse trials were also designed to evaluate the combination of 1,3-D and MNa as an alternative to MB for cucumber production in China.

## Materials and methods

### Laboratory studies

The efficiency of 1,3-D and MNa was observed with sole and combined applications in the laboratory experiment. Soil samples were collected randomly at 0–20 cm depth from a cucumber greenhouse in Shunyi, Beijing, known to have severe contamination of soil-borne pests. Soil sample analysis revealed that the soil contained 20.34% sand, 76.25% silt, and 3.41% clay with 35.64 g kg^-1^ soil of organic matter contents and pH 6.40. The soil samples were prepared by sieving through a 2-mm mesh followed by thorough mixing. The soil moisture content was 23.80% (w/w). The pH was measured by preparing extracts at a ratio of 1:2.5 (soil:H_2_O). The moisture content of the soil was determined by heating it in a drying oven at 105 ± 5°C for 6 hours.

A total of 600 g soil was placed in 27 2.5-L desiccators. Ten *A*. *theophrasti* seeds and fifteen *D*. *sanguinalis* seeds were sown at a depth of 2 cm in each desiccator [27]. Three replicates were performed for each treatment: 1,3-D alone (10, 20 mg a.i. kg^-1^ soil), MNa alone (20, 40 mg a.i. kg^-1^ soil), 1,3-D plus MNa (10+20, 10+40, 20+20, 20+40 mg a.i. kg^-1^ soil) and the control. 1,3-D or MNa was respectively pipetted into the soil (Eppendorf, Germany), after which the desiccators were immediately sealed. The desiccators were utilized for 5 days at 28°C, when, for one day, the covers were removed to allow residual fumigant to exhaust; then, the weed height was determined. *F*. *oxysporum* was quantitatively isolated from the fumigated soil as described by Komada [28]. A sub-soil sample of 100g was used to isolate root-knot nematodes (*M*. *incognita*) as described by Liu [29].

### Greenhouse trials

In 2012 and 2013–2014, with the authorization of the Beijing Agriculture Bureau, two demonstration experiments were carried out in greenhouse growing cucumbers (*C*. *sativus* cv. “Zhongnong No.16”, Beijing, China) on a commercial farm in Tongzhou, Beijing (trial I: 39°53’59.5”N, 116°44’46.4”E) and cucumbers (*C*. *sativus* cv. “Jinyou No.35”, Tianjin, China) in Shunyi, Beijing (trial II: 40°03’11.4”N, 116°55’51.4”E), which are considered to be the core areas for the cultivation of this vegetable. The greenhouse study site did not involve endangered or protected species. Both farms are facing complications caused by heavily infectious root-knot nematodes, some soil-borne fungi, and other pests. Further details of this experiment are shown in Tables 1 and 2.

**Table 1:** Soil characteristics of the experimental sites.

Site	Soil moisture (%)	pH (1:2.5)	Organic matter (g kg^-1^)	N/NH_4_^+^ (mg kg^-1^)	N/NO_3_^-^ (mg kg^-1^)	Available K (mg kg^-1^)	Available P (mg kg^-1^)	Bulk density (g·cm^-3^)
Tongzhou, 2012	18.50	7.07	33.0	37.2	233.7	815.3	318.6	0.95
Shunyi, 2013–2014	22.23	6.85	37.7	2.9	69.4	1170.6	532.3	0.86

**Table 2:** Relevant greenhouse trial dates and additional details.

Site	Cucumber cultivar	Block area (m^2^)	Fumigant application	Tarp removal	Cucumber transplant	End of the trial	Last cropapp:ds:preceding crop
Tongzhou, 2012	No.16 Zhongnong	3.2×5.7 = 18.24	05/08/12; 13/08/12	27/08/12	05/09/12	25/11/12	Tomato
Shunyi, 2013–2014	No.35 Jinyou	3.6×5.6 = 20.16	10/09/13; 16/09/13	26/09/13	05/11/13	28/05/14	Cucumber

For the treatments, 1,3-D 95 EC (Hunan Yueyang Yunxi Daorenji Solvent Chemical Factory, China, containing 95% 1,3-D), MNa 42% AS (Shenyang Harvest Agrochemical Co., Ltd., China, a marketable product containing 42% MNa) and MB 98 TC (Changyi Chemical Plant, China, containing 98% MB) were used. The soil was mulched with polyethylene film (0.04 mm thick, Baoding Baoshuo Plastic Co., Ltd., Hebei Province, China).

All treatments were performed in randomized blocks including three replicates (Table 3). MB was used as the reference treatment. Experimental treatments included applications of 1,3-D alone, MNa alone, the combination of 1,3-D and MNa, and an untreated control. Chemigation with 1,3-D was performed at a rate of 20 g a.i. m^-2^, and MNa was applied by chemigation at a rate of 40 g a.i. m^-2^. The treatment of 1,3-D combined with MNa was applied as follows: MNa was first applied by chemigation at a rate of 20 g a.i. m^-2^. Approximately one week later, 1,3-D was applied by chemigation at a rate of 10 g a.i. m^-2^. The MB was applied utilizing the hot gas method at 50 g m^-2^. These treatments were enclosed in a PE film.

**Table 3:** Experimental treatments in two greenhouse trials.

Sites	Treatment^a^	Rate (g a.i. m^-2^)	Tarp kind^b^	Application methods	Fumigation date
Tongzhou, 2012	1,3-D 95 EC	20	PE	Chemigation	05/08/12
	MNa 42 AS	40	PE	Chemigation	05/08/12
	1,3-D 95 EC+MNa 42 AS	10+20	PE	Chemigation	MNa: 05/08/12; 1,3-D: 13/08/12
	MB 98 TC	50	PE	Gas distribution	13/08/12
	Untreated	/	/	/	/
Shunyi, 2013–2014	1,3-D 95 EC	20	PE	Chemigation	10/09/13
	MNa 42 AS	40	PE	Chemigation	10/09/13
	1,3-D 95 EC+MNa 42 AS	10+20	PE	Chemigation	MNa: 10/09/13; 1,3-D: 16/09/13
	MB 98 TC	50	PE	Gas distribution	10/09/13
	Untreated	/	/	/	/

^a^ Abbreviations: 1,3-D = 1,3-dichloropropene; MNa = metam sodium; MB = methyl bromide; EC = emulsifiable concentrate; AS = aqueous solution; TC = technical.

^b^ Abbreviations: PE = polyethylene film.

A data logger (XR440 Pocket Data logger, Pace Scientific, United States) was used to measure the soil temperature at a 15-cm depth only in the plot where 1,3-D and MNa were applied together during the soil fumigation period.

The population of the soil-borne fungus [colony-forming units (cfu) g^-1^ soil] was assessed from the fumigated soil at a depth of 0–20 cm. Soil from each plot was sampled from 3 different locations along diagonal lines. The population density of the soil root-knot nematode was estimated after fumigation to a depth of 0–20 cm. The soil was sampled as described above. Soil-borne fungi and root-knot nematodes were assessed utilizing the same methods as in the laboratory studies.

The height of the plants was noted 4 weeks after the crop was transplanted (WAT) (20 plants per plot). Root galls and the severity of root disease in cucumber were both assessed at the end of the trials (20 plants/plot). Root galls were rated on a 0–4 scale indicating the percentage of root galls: 0 = 0%, 1 = 1–25%, 2 = 26–50%, 3 = 51–75%, and 4 = 76–100% [3]. Root disease severity was classified based on a severity scale as follows: 0 = healthy plants and roots without disease; 1 = rotten roots with blackish brown color compose <25% of the root system; 2 = 26–50%; 3 = 51–75%; and 4 = 76–100% black-brown rotten roots [30]. The fruit yield and income generated by the crops were recorded after each harvest and summarized upon completion of the research experiment.

### Statistical analyses

#### Laboratory studies

The mortality rate of the nematodes was quantified utilizing the following equation.

$$X=\frac{N_{1}}{N_{1}+N_{2}}\times 100$$ (1)

where *X* indicates the mortality rate for nematode (%), *N*_*1*_ indicates the number of nematodes that died during experiment, and *N*_*2*_ indicates the number of nematodes that remained alive.

The correction applied to the nematode mortality calculation was performed with the following equation:

$$Y=\frac{X_{1}-X_{2}}{1-X_{2}}\times 100$$ (2)

where *Y* = corrected nematode mortality rate (%), *X*_*1*_ = mortality of nematodes for fumigated treatments (%), and *X*_*2*_ = nematode mortality in the control treatment(%).

The efficiency of weed and fungal control was calculated with the following equation.

$$Y=\frac{X_{1}-X_{2}}{X_{1}}\times 100$$ (3)

where *Y* is the efficiency against weeds or fungus, *X*_*1*_ indicates the height of the weeds and the fungal populations present in the control, and *X*_*2*_ is the fungal populations or weed height in fumigant-treated plots.

The control efficacy for the combined application of 1,3-D and MNa was identified as follows [31]:

$$E_{0}=\frac{X_{1}\times X_{2}}{100}$$ (4)

where *E*_*0*_ is the expected control efficacy for the combination, *X*_*1*_ is the measured control efficacy for one fumigant, and *X*_*2*_ is the measured control efficacy depending on another fumigant. *E* is the measured control efficacy for combined application. If *E* < *E*_*0*_, the combination was antagonistic; if *E* > *E*_*0*_, the combination was complementary.

#### Greenhouse trials

The efficacy of controlling nematode or fungi was calculated using Eq (3).

The calculation for the root galling or root disease index was determined with the following equation:

$$Y=\frac{R_{1}+R_{2}+\ldots +R_{x}}{r\times x}\times 100$$ (5)

where *Y* indicates the root galling index or root disease index, *Rx* is the root galling scale or root disease scale for the *x*-th plant, *r* is the maximum root galling scale or root disease scale, and *x* is the total number of plants.

For statistical analysis, the results collected for the soil fungal and nematode populations were converted as required (square root transformations for small numbers [<100] and log10 for large numbers [>100]). However, all of the data are reported as untransformed values. The data were analyzed with SAS statistical techniques for ANOVA (SAS, version 8.0 for Windows). Significantly different mean values were calculated using Fisher’s LSD test with *P* = 0.05.

## Results

### Laboratory studies

#### Root-knot nematodes

Among the four fractions tested, the rates of the combined treatment of 1,3-D+MNa (10 + 20 mg a.i. kg^-1^ soil) resulted in a strong reduction in the population of root-knot nematodes (E-E_0_ = 13.68), reducing the *M*. *incognita* numbers by 89.10% (Table 4).

**Table 4:** Laboratory studies on the 1,3-D+MNa combination on root-knot nematodes, soil-borne fungus and weed seeds.

Treatment^a^	Rate (g a.i. kg^-1^ soil)	% Corrected nematode mortality				%Control efficacy on *Fusarium oxysporum*				%Control efficacy on *Abutilon theophrasti*				%Control efficacy on *Digitaria sanguinalis*			
		*E*^b^	*E*_*0*_^c^	*E*- *E*_*0*_	CE^d^	*E*	*E*_*0*_	*E*- *E*_*0*_	CE	*E*	*E*_*0*_	*E*- *E*_*0*_	CE	*E*	*E*_*0*_	*E*- *E*_*0*_	CE
1,3-D	10	38.14				50.39				32.07				29.52			
1,3-D	20	80.45				71.89				49.49				65.18			
MNa	20	60.26				56.22				42.41				11.64			
MNa	40	84.87				59.94				100.00				56.30			
1,3-D+MNa	10+20	89.10	75.42	13.68	+	80.96	78.28	2.68	+	97.70	60.88	36.82	+	70.82	37.72	33.10	+
1,3-D+MNa	10+40	91.03	90.64	0.39	+	82.22	80.13	2.09	+	100.00	100.00	0.00	±	94.54	69.20	25.34	+
1,3-D+MNa	20+20	91.35	92.23	-0.88	-	87.49	87.69	-0.20	-	100.00	70.91	29.09	+	86.08	69.23	16.85	+
1,3-D+MNa	20+40	93.91	97.04	-3.13	-	90.86	88.74	2.12	+	100.00	100.00	0.00	±	98.78	84.78	14.00	+
Untreated																	

^a^ Abbreviations: 1,3-D = 1,3-dichloropropene; MNa = metam sodium.

^b^ Abbreviations: *E* = the measured control efficacy of the combination.

^c^ Abbreviations: *E*_*0*_ = the expected control efficacy of the combination.

^d^ Abbreviations: CE = combined efficacy. If *E*—*E*_*0*_ > 0, CE was expressed as +; if *E*—*E*_*0*_ < 0, CE was expressed as -; if *E*—*E*_*0*_ = 0, CE was expressed as ±.

#### Soilborne fungus

All the rates of 1,3-D+MNa (except for 20+20 mg a.i. kg^-1^ soil) examined successfully reduced the concentrations of *F*. *oxysporum* (Table 4). The levels of *F*. *oxysporum* were reduced by at least 80.96%. The highest complementary efficacy was observed in the 1,3-D+MNa 10+20 mg a.i. kg^-1^ treatment (E-E_0_ = 2.68).

#### Weeds

All four 1,3-D+MNa rates tested were found to reduce the population of *D*. *sanguinalis* (Table 4). The 1,3-D+MNa 10+20 mg a.i. kg^-1^ treatment provided the strongest complementary effects on both *A*. *theophrasti* (97.70%, E-E_0_ = 36.82) and *D*. *sanguinalis* (70.82%, E-E_0_ = 33.10).

### Greenhouse trials

#### Soil temperature

During the fumigation, the temperature of the soil at a 15-cm depth remained at 20.3–30.1°C under trial II in the 1,3-D+MNa plots.

#### Root-knot nematodes

The untreated controls in trial I (in 2012) and trial II (in 2013–2014) were both heavily infested with *M*. *incognita* (Table 5). During the two greenhouse trials, significantly higher levels of *M*. *incognita* were observed in the untreated control group than those in all fumigant treatments except for MNa used alone in trial II, 2013–2014.

**Table 5:** Effects of soil fumigation on the numbers of *Meloidogyne* spp. recovered from 100 g of soil and colony-forming units (cfu) of *Fusarium oxysporum* on selective media of 1 g from soil after fumigation.

Site	Treatment^a^	Rate (g a.i. m^-2^)	Tarp kind^b^	*Meloidogyne incognita*		*Fusarium oxysporum*	
				No. 100 g^-1^^c^	% reduction	cfu g^-1^	% reduction
Tongzhou, 2012	1,3-D	20	PE	0c^c^	100.00	1122ab	48.80
	MNa	40	PE	3b	98.74	320bc	85.39
	1,3-D+MNa	10+20	PE	0c	100.00	165c	92.49
	MB	50	PE	0c	100.00	11d	99.49
	Untreated	/	/	258a	/	2191a	/
Shunyi, 2013–2014	1,3-D	20	PE	18b	94.38	1368b	74.01
	MNa	40	PE	100ab	69.23	1233b	76.59
	1,3-D+MNa	10+20	PE	28b	91.54	532bc	89.90
	MB	50	PE	14b	95.62	367c	93.03
	Untreated	/	/	325a	/	5265a	/

^a^ Abbreviations: 1,3-D = 1,3-dichloropropene; MNa = metam sodium; MB = methyl bromide.

^b^ Abbreviations: PE = polyethylene film.

^c^ In each column, data are means of three replications. Means followed by the same letter are not different (*P* = 0.05) according to the LSD test.

During trial I, 2012, the treatment combination of 1,3-D+MNa decreased the levels of nematodes by 100.00%, which was the same statistically as the treatments with MB or 1,3-D alone, but was significantly lower than those with MNa alone. Similarly, in trial II, 2013–2014, the treatment combination of 1,3-D+MNa decreased the nematode levels by 91.54%, which was not significantly different from the effects of the other chemical treatments. The difference in nematode control was not significant between MNa used alone and the untreated control.

#### Soil-borne fungus

The untreated control group was infested heavily by both *F*. *oxysporum* in both trials (Table 5). Significantly higher levels of *F*. *oxysporum* were found in the untreated control group than those in all of the fumigant treatments with the exception of the sole use of 1,3-D in trial I, 2012 (Table 5).

*F*. *oxysporum* was reduced in all the chemical treatments by at least 85.39% and 76.59% in trial I and trial II, except for the 1,3-D treatment (Table 5). In the two greenhouse trials, the lowest *F*. *oxysporum* level was observed in the MB treated plot, followed by 1,3-D+MNa, MNa, 1,3-D, and the untreated control.

#### The first cucumber fruit yield

Fumigated treatments affected the first cucumber fruit yield (Table 6). The first fruit yield in both trials was the lowest (0.04 and 0.13 kg m^-2^, respectively) in the untreated plots of cucumbers (Table 6). In trial I, the 1,3-D-treated plots had the highest yield (0.24 kg m^-2^), although statistically, the difference was not significant when compared to the other chemical treatments except that the level of control was significantly higher than that in the untreated control (Table 6). In trial II, the highest yield of cucumbers was observed in the MB-treated plot (0.31 kg m^-2^), but this value was not significantly different from those of the other chemical treatments; however, the first yields provided by all the chemical treatments were significantly higher than those of the untreated control (Table 6).

**Table 6:** Effect of fumigation programs on the first cucumber yield.

Site	Date	Price (¥ kg^-1^)	Treatment^a^	Rate (g a.i. m^-2^)	Yield (kg m^-2^)
Tongzhou, 2012	05/10/12	2.2	1,3-D	20	0.24a^b^
			MNa	40	0.08ab
			1,3-D+MNa	10+20	0.20ab
			MB	50	0.19ab
			Untreated	/	0.04b
Shunyi, 2013–2014	09/12/13	2.0	1,3-D	20	0.28a
			MNa	40	0.27a
			1,3-D+MNa	10+20	0.27a
			MB	50	0.31a
			Untreated	/	0.13b

^a^ Abbreviations: 1,3-D = 1,3-dichloropropene; MNa = metam sodium; MB = methyl bromide.

^b^ In the column, data are means of three replications. Means followed by the same letter do not differ significantly (*P* = 0.05) according to the LSD test.

#### Cucumber plant height, root galling index, root disease index, yield and income

In the two cucumber greenhouse trials, the plant height at 4 WAT was significantly affected in the fumigation treatments (Table 7). The height of the cucumber plants was significantly higher in all the chemical treatments when compared to that of the untreated control group. No significant difference was observed between the fumigant treatments in both trials except for 1,3-D treatment in trial II (Table 7).

**Table 7:** Effect of fumigation programs on cucumber plant height, root galling index, root disease index, yield and income.

Site	Treatment^a^	Rate (g a.i. m^-2^)	Plant height^b^ (cm)	Root galling index^c^ (%)	Root disease index (%)	Yield^d^ (kg m^-2^)	Income^e^ (¥ m^-2^)
Tongzhou, 2012	1,3-D	20	204.4a^f^	2.04c	0.56c	3.96ab	9.32ab
	MNa	40	189.4a	31.11b	7.50b	3.23b	7.81b
	1,3-D+MNa	10+20	205.4a	6.20c	2.59c	4.12ab	9.40a
	MB	50	209.7a	0.37c	0.00c	4.91a	10.51a
	Untreated	/	141.3b	42.78a	17.22a	2.25c	5.86c
Shunyi, 2013–2014	1,3-D	20	174.6b	11.67c	15.00bc	9.39ab	21.88ab
	MNa	40	181.8ab	39.17b	30.83b	8.79b	20.64b
	1,3-D+MNa	10+20	179.3ab	5.00c	5.00cd	9.92a	24.39ab
	MB	50	186.1a	3.33c	4.17d	10.12a	25.56a
	Untreated	/	156.3c	78.33a	63.33a	7.66c	14.04c

^a^ Abbreviations: 1,3-D = 1,3-dichloropropene; MNa = metam sodium; MB = methyl bromide.

^b^ Cucumber height collected at 4 WAT.

^c^ Root galling index and root disease index were collected at the end of the trials.

^d^ Cucumber yield collected at each harvest time and summed together at the end of the trials.

^e^ Cucumber income collected at each harvest time and accumulated together at the end of the trials.

^f^ In each column, data are means of three replications. Means followed by the same letter are not significantly different (*P* = 0.05) according to the LSD test

Infestation by nematodes at the end of the trials was estimated by computing the root galling index. The highest root galling index (42.78% and 78.33% in trials I and II, respectively) both occurred in the untreated plots. In the two greenhouse trials, the lowest root galling indices were both observed in the MB-treated plot but did not differ significantly from the 1,3-D+MNa or the 1,3-D treatments. The previous three fumigant treatments were significantly more effective than the MNa treatment. The root galling index of each fumigant treatment was significantly lower than that of the untreated controls in two trials (Table 7).

The root disease index was significantly affected in the fumigation treatments. The untreated plots had the highest root disease indices (17.22% and 63.33% in trials I and II, respectively). In trial I, the plots treated with MB had the lowest index (0.00%), but it was not significantly different from the 1,3-D+MNa or 1,3-D treatments. The previous three fumigant treatments provided significantly better results than the MNa treatment. In trial II, the lowest index (4.17%) was obtained in the MB-treated plots, and it was significantly lower than all the other treatments except for the 1,3-D+MNa treatment.

The yield of cucumbers was significantly affected in the chemical treatments (Table 7). The untreated plots of cucumbers provided the lowest yield (2.25 and 7.66 kg m^-2^, respectively, in trials I and II) compared to the chemical treatments. In trial I, the highest yield (4.91 kg m^-2^) in plots with the MB treatment was observed, but it dit not differ from treatments with 1,3-D+MNa or 1,3-D alone. However, this value was significantly higher than the plots treated solely with MNa. The highest yield (10.12 kg m^-2^) in trial II was also observed in the plots treated with MB, but the value was not significantly different from the treatments with 1,3-D+MNa or 1,3-D alone; however, itwas significantly higher than the yield in the sole treatment with MNa.

The farmers’ incomes from the production of cucumbers varied among treatments (Table 7). Compared with the chemical treatments, the lowest income was achieved with the untreated cucumbers in both trials (5.86 and 14.04 ¥ m^-2^, respectively). The MB-treated plots provided the highest income (10.51 and 25.56 ¥ m^-2^, respectively) in both trials. Neither of these values differed significantly from those of 1,3-D+MNa or 1,3-D alone but were significantly higher than those from plots treated solely with MNa.

## Discussion

1,3-D has proved to be as effective as MB in controlling root-knot nematode globally [3,5,7], and the present laboratory studies and greenhouse trials confirmed this result. However, most formulations of 1,3-D have poor efficacy against weeds and soil-borne fungi. Thus, additional fungicide and herbicide requirements are necessary to expand the control of weeds and soil-borne fungi. MNa, an MITC generator, could effectively control soil-borne fungi and weeds. The laboratory studies indicated that MNa was highly effective against weeds, and similarly, normal activity was observed for soil-borne fungi. Therefore, the combined use of 1,3-D and MNa as a broad-spectrum soil fumigant was at least theoretically feasible. Laboratory studies showed that the combination of 1,3-D and MNa (10+20 mg a.i. kg^-1^ soil) had the best complementary effects on root-knot nematodes, soil-borne fungi, and the seeds of two important weed species. The specific synergistic mechanism of 1,3-D and MNa remained unclear in this study, though the laboratory results gave a preliminarily indication of the feasibility of their combined application and confirmed the synergistic activity of combinations of 1,3-D (+ Pic) and MNa reported by Desaeger *et al*. [3].

The combined application of 1,3-D and MNa in the greenhouse at a rate of 10 + 20 g a.i. m^−2^ showed a significant ability to suppress *M*. *incognita* root galling, minimize cfu of *F*. *oxysporum* efficiently on growth media, and promote high cucumber yields. These results were not significantly different from those of the sole application of MB at 50 g m^−2^ or 1,3-D at 20 g m^−2^, but the results were significantly better than those achieved when MNa was applied alone at a level of 40 g m^−2^. The field results of the experiment also confirmed that combinations of 1,3-D and MNa are effective alternatives to MB.

True mixtures of these two fumigants are presently not available due to the chemical incompatibility in their combination [3,23,24]. In the laboratory, antagonistic effects observed in some high dose combinations of 1,3-D and MNa could be attributed to their degradation (Table 4). Desaeger *et al*. found that combining MNa and 1,3-D+Pic in a tank to mix together for chemigation induced the rapid generation of cloudy, black material that could potentially clog the drip emitters, revealing that the combination of 1,3-D and MNa should not be applied for chemigation simultaneously [3]. In the present greenhouse trials, MNa was applied approximately one week before 1,3-D to minimize the degradability of the latter and improve the control of root-knot nematodes. Machinery that allows the injection of two fumigants independently without contact during the application to reduce negative side effects has been developed in Australia [32].

In summary, the combination of the fumigants 1,3-D and MNa was almost as effective as MB at controlling soil-borne nematodes and fungi while maintaining high cucumber yields and the income of farmers. However, more detailed research is required to identify the optimal application protocols—including formulations and appropriate combinations with biological agents such as *Bacillus subtilis* [33] or resistance-inducing agents such as acibenzolar-S-methyl [34]—before the combination of 1,3-D and MNa can be recommended as an effective substitute for MB for sustainable cucumber production in China.
